# Identification of Two Novel Peptides That Inhibit α-Synuclein Toxicity and Aggregation

**DOI:** 10.3389/fnmol.2021.659926

**Published:** 2021-04-12

**Authors:** Blagovesta Popova, Dan Wang, Abirami Rajavel, Karthikeyan Dhamotharan, Diana F. Lázaro, Jennifer Gerke, Joachim F. Uhrig, Michael Hoppert, Tiago F. Outeiro, Gerhard H. Braus

**Affiliations:** ^1^Department of Molecular Microbiology and Genetics, Institute for Microbiology and Genetics, University of Goettingen, Göttingen, Germany; ^2^Department of Experimental Neurodegeneration, Center for Biostructural Imaging of Neurodegeneration, University Medical Center Goettingen, Göttingen, Germany; ^3^Department of Plant Molecular Biology and Physiology, University of Goettingen, Göttingen, Germany; ^4^Department of General Microbiology, Institute of Microbiology and Genetics, University of Goettingen, Göttingen, Germany; ^5^Max Planck Institute for Experimental Medicine, Göttingen, Germany; ^6^Translational and Clinical Research Institute, Faculty of Medical Sciences, Newcastle University, Framlington Place, Newcastle upon Tyne, United Kingdom; ^7^Deutsches Zentrum für Neurodegenerative Erkrankungen (DZNE), Göttingen, Germany

**Keywords:** α-synuclein, Parkinson’s disease, protein aggregation, oligomerization, peptide drug discovery, yeast, library screening

## Abstract

Aggregation of α-synuclein (αSyn) into proteinaceous deposits is a pathological hallmark of a range of neurodegenerative diseases including Parkinson’s disease (PD). Numerous lines of evidence indicate that the accumulation of toxic oligomeric and prefibrillar αSyn species may underpin the cellular toxicity and spread of pathology between cells. Therefore, aggregation of αSyn is considered a priority target for drug development, as aggregation inhibitors are expected to reduce αSyn toxicity and serve as therapeutic agents. Here, we used the budding yeast *S. cerevisiae* as a platform for the identification of short peptides that inhibit αSyn aggregation and toxicity. A library consisting of approximately one million peptide variants was utilized in two high-throughput screening approaches for isolation of library representatives that reduce αSyn-associated toxicity and aggregation. Seven peptides were isolated that were able to suppress specifically αSyn toxicity and aggregation in living cells. Expression of the peptides in yeast reduced the accumulation of αSyn-induced reactive oxygen species and increased cell viability. Next, the peptides were chemically synthesized and probed for their ability to modulate αSyn aggregation *in vitro*. Two synthetic peptides, K84s and K102s, of 25 and 19 amino acids, respectively, significantly inhibited αSyn oligomerization and aggregation at sub-stoichiometric molar ratios. Importantly, K84s reduced αSyn aggregation in human cells. These peptides represent promising αSyn aggregation antagonists for the development of future therapeutic interventions.

## Introduction

Protein misfolding and aggregation is a hallmark event in a growing number of human diseases, including Parkinson’s disease (PD). PD is the second most common neurodegenerative disorder, affecting 1–2% of the population over the age of 60. The pathological hallmark of the disease is a massive loss of dopaminergic neurons in the *substantia nigra pars compacta* of the brain (German et al., 1992). PD is characterized by the deposition of the small neuronal protein α-synuclein (αSyn) into intracellular inclusions, known as Lewy bodies (LB; Spillantini et al., 1997). Missense mutations and genomic multiplications of αSyn gene (*SNCA*) are linked to autosomal dominant familial PD, which marks αSyn as a prime target in PD research (Polymeropoulos et al., 1997; Krüger et al., 1998; Singleton et al., 2003; Chartier-Harlin et al., 2004; Zarranz et al., 2004; Appel-Cresswell et al., 2013; Lesage et al., 2013). The αSyn protein is intrinsically disordered and can self-assemble into oligomeric protofibrils that can further mature into different types of fibrils and aggregates (Breydo et al., 2012). Pathological conditions promote αSyn aggregation, especially in connection with genetic mutations (Conway et al., 1998; Fredenburg et al., 2007), molecular crowding (Shtilerman et al., 2002; Uversky et al., 2002), increased αSyn protein levels (Conway et al., 1998; Breydo et al., 2012), post-translational modifications (Stefanis, 2012; Popova et al., 2015), low pH (Ahmad et al., 2012), or oxidative conditions (Hashimoto et al., 1999). *in vitro* studies suggest that αSyn exists in various conformations and oligomeric states in a dynamic equilibrium, where the monomer can aggregate into small oligomeric species, stabilized by β-sheet interactions, which slowly convert into higher molecular weight insoluble protofibrils and amyloidogenic fibrils, resembling those found in LB (Conway et al., 1998, 2000; Karpinar et al., 2009; Bengoa-Vergniory et al., 2017). The mechanisms promoting pathological protein aggregation are still unknown. There is a substantial and increasing body of evidence implicating accumulation of oligomeric/protofibrillar αSyn species as one major contribution to neurodegeneration (Karpinar et al., 2009; Winner et al., 2011; Bengoa-Vergniory et al., 2017). The mechanism of αSyn oligomer-induced neurotoxicity involves disruption of numerous cellular processes, among them, are increased mitochondrial, lysosomal, and vesicular membrane permeability, autophagic and lysosomal dysfunction, proteasomal effects, endoplasmic reticulum stress and synaptic dysfunction (Bengoa-Vergniory et al., 2017). Several studies demonstrated that certain species of αSyn can seed the aggregation and formation of inclusions both *in vivo* and *in vitro* (Hansen et al., 2011; Luk et al., 2012). Therefore, inhibition of αSyn aggregation is an extremely important target for drug development. Inhibitors that prevent oligomerization and aggregation of the protein are expected to serve as therapeutic medicines that can prevent the propagation of the disease (Dehay et al., 2015).

We and others have demonstrated that complex diseases such as PD can be modeled in a simple eukaryotic organism such as the budding yeast *Saccharomyces cerevisiae* due to the high conservation of biological pathways, affected by protein misfolding and aggregation (Outeiro and Lindquist, 2003; Cooper et al., 2006; Gitler et al., 2008; Petroi et al., 2012; Lázaro et al., 2014; Kleinknecht et al., 2016; Popova et al., 2021). Strikingly, although there is no *SNCA* homolog in the yeast genome, the expression of the protein in yeast recapitulates several relevant aspects of PD. Overexpression of αSyn results in growth impairment and accumulation of the protein into cytoplasmic inclusions similar to the pathogenesis of the disease (Outeiro and Lindquist, 2003; Petroi et al., 2012). Therefore, yeast emerges as a powerful platform for primary screening for cytoprotective compounds (Tenreiro et al., 2017).

In this study, we used a peptide library and performed two high-throughput screens to isolate peptides that reduce αSyn-associated toxicity and aggregation. From a pool of one million candidates, we isolated seven candidates that specifically reduce the toxicity and aggregation of human αSyn in yeast. Two peptides with a length of 25 amino acids (K84s) and 19 amino acids (K102s) are effective inhibitors of αSyn oligomerization and fibrilization as determined with Thioflavin-T staining of amyloid fibrils, electron microscopy imaging, and biochemical analysis. Importantly, K84s reduced αSyn aggregation in the human cell.

## Materials and Methods

### Yeast Strains, Transformation and Growth Conditions

Plasmids and *Saccharomyces cerevisiae* strains are listed in Table 1 and Table 2. Yeast plasmids were constructed using GENEART Seamless cloning and assembly kit (Life technologies). All constructs were verified by DNA sequencing. Yeast strains were grown in YPAD (Yeast Extract—Peptone—Dextrose plus Adenine medium) or synthetic complete dropout (SC) medium (Guthrie and Fink, 1990), lacking the respective amino acid for selection, supplemented with 2% glucose, 2% raffinose or 2% galactose. *S. cerevisiae* strains were used for transformations performed by standard lithium acetate procedure (Gietz et al., 1992).

**Table 1:** Plasmids used in this study.

Name	Description	Source
pGADT7	*2μ; LEU2; ADH1pr; ADH1term; AmpR*	Clontech Inc.
pGBKT7	*2μ; TRP1; ADH1pr; ADH1term; KanR*	Clontech Inc.
pME4897	*pGBKT7-GAL4-BD-SNCA*	This study
pME4898	*pGADT7-AD-K50*	This study
pME4899	*pGADT7-AD-K84*	This study
pME4900	*pGADT7-AD-K102*	This study
pME4901	*pGADT7-AD-K89*	This study
pME4902	*pGADT7-AD-K117*	This study
pME4903	*pGADT7-AD-K94*	This study
pME4904	*pGADT7-AD-K97*	This study
p425-GPD	*2μ; LEU2; GPDpr; CYC1term; AmpR*	Mumberg et al. (1994)
pME4905	*p425-GPD-K50*	This study
pME4906	*p425-GPD-K84*	This study
pME4907	*p425-GPD-K94*	This study
pME4908	*p425-GPD-K89*	This study
pME4909	*p425-GPD-K97*	This study
pME4910	*p425-GPD-K102*	This study
pME4911	*p425-GPD-K117*	This study
p424-GAL1	2 μ*m; TRP1; GAL1pr; CYC1term; AmpR*	Mumberg et al. (1994)
p425-GAL1	2 μ*m; LEU2; GAL1pr; CYC1term; AmpR*	Mumberg et al. (1994)
p426-GAL1	2 μ*m; URA3, GAL1pr; CYC1term; AmpR*	Mumberg et al. (1994)
pME3759	*p426-GAL1-GFP*	Petroi et al. (2012)
pME3772	*p426-GAL1-SNCA-mCherry*	Petroi et al. (2012)
pRS304	*TRP1; GAL1pr; CYC1term; AmpR*	Sikorski and Hieter (1989)
pME3597	*pRS304-GAL1-SNCA*	Shahpasandzadeh et al. (2014)
Addgene 15587	*p303-GAL1-FLAG-htt103QΔPro-CFP*	Duennwald et al. (2006)
pME4912	*pET22b-K50–6xHis*	This study
pME4913	*pET22b-SNCA*	This study

**Table 2:** Yeast strains used in this study.

Name	Genotype	Source
W303-1A	*MATa, ura3–1, trp1–1, leu2–3_112, his3–11, ade2–1, can1–100*	EUROSCARF
BY4741	*MATa, ura3Δ0, his3Δ 1, leu2Δ0, met15Δ0*	EUROSCARF
RH3465	W303 containing *GAL1-GFP* in *ura3* locus	Petroi et al. (2012)
RH3468	W303 containing three genomic copies of *GAL1-SNCA-GFP* in *ura3* locus	Petroi et al. (2012)
AH109	*MATa, trp1–901, leu2–3, 112, ura3–52, his3–200, gal4Δ, gal80Δ, LYS2::GAL1UAS-GAL1TATA-HIS3, GAL2UAS-GAL2TATA-ADE2, URA3 : : MEL1UAS-MEL1TATA-lacZ, MEL1*	Clontech Inc.
Y187	*MATalpha, ura3–52, his3–200, ade2–101, trp1–901, leu2–3, 112, gal4Δ, met–, gal80Δ, URA3 : GAL1UAS-GAL1 TATA-lacZ, MEL1*	Clontech Inc.
RH3788	BY4741 *containing GAL1-FLAG-htt103QΔPro-CFP* in *his3* locus	This study
AH109-syn	AH109 containing *GAL1-SNCA* in *trp1* locus	This study

### Spotting Assay

For growth test on solid medium, yeast cells were pre-grown in minimal medium containing 2% raffinose lacking the corresponding marker to mid-log phase. Cells were normalized to equal densities, serially diluted 10-fold starting with an OD_600_ of 0.1, and spotted on SC-plates containing either 2% glucose or 2% galactose and lacking in the corresponding marker. The plates were incubated at 30°C for 3 days. Singer ROTOR HDA bench robot (Singer Instruments, UK) was used for robotic pinning from a 96-well liquid cell suspension (source plate) onto multiple solid agar (target) plates using the manufacturer’s software.

### Growth Analysis in Liquid Culture

Cells were pre-grown in 2% raffinose-containing selective SC medium until logarithmic growth phase and inoculated in 2% galactose-containing SC medium to equal densities of OD_600_ = 0.1. Optical density measurements of 100 μl cell cultures were performed in quadruplicates in 96-well plates for 24 h using a microplate reader with temperature control and continuous shaking (Infinite M200, Tecan).

### Yeast-Two-Hybrid Screen

The library approach of yeast two-hybrid high-throughput screening involves a bait protein being screened against a pool of prey proteins. Yeast clones expressing interacting proteins are selected based on reporter gene activation and the resulting ability to grow on selective media. The yeast strains AH109 (Mat a) and Y187 (Mat α) were used for the Y2H experiments. The strain AH109 was transformed with the *GAL4*-*BD*-*SNCA* bait construct (pME4897). The random peptide library (four million primary clones) was obtained from Fritz and Green (Fritz and Green, 1996) and amplified by transformation in *E. coli*. Plasmid was prepared in bulk from a pool of one million individually grown colonies and transformed into Y187 strain (prey constructs). To warrant a sufficiently complete library representation after the transformation of yeast strain Y187, five million individual prey-containing yeast colonies have been obtained, pooled, and frozen in aliquots. Aliquots were used for mating with a freshly prepared bait yeast strain. The AH109 strain, transformed with the bait construct was inoculated in 50 ml SC-Trp from a fresh single colony overnight. On the next day, one library aliquot was thawed at 37°C for 10 min and mixed with 9 ml YPAD medium to regenerate for 2 h at 30°C. The optical density of both strains was measured at 600 nm after appropriate dilution. 10 OD_600_ units from both strains were pelleted and mixed with 10 ml YPAD containing 20% PEG6000. Mating of the cells was achieved by incubating the flask at 30°C for 6–8 h at 80 rpm on a rotation shaker. After successful mating, the cells were pelleted and resuspended gently in 2 ml SC-Leu-Trp-His liquid medium containing 2% glucose. Mating efficiency was checked by plating of 10 μl, 20 μl, and 50 μl inoculum on SC-Leu-Trp plates (selection for diploids) to obtain >5 million zygotes in each screening experiment ensuring complete representation of the library. The rest of the cell suspension was used to inoculate 500 ml of SC-Leu-Trp-His Semi-Solid Media (SSM), containing 1% Gelrite, 2% glucose, and supplemented with ampicillin. The SSM containing the diploid cells was then mixed vigorously using a magnetic stirrer, equally distributed among 30 Petri dishes, and incubated at 30°C for 5–10 days. Colonies observed on SC-Leu-Trp-His SSM were collected by transferring 10 μl of the grown single colony using a pipette into a 96-well plate containing 90 μl of SC-Leu-Trp-His liquid media. The cells were resuspended by mixing and incubated at 30°C overnight. The 96-well plates were spotted onto solid SC-Leu-Trp agar plates using Singer ROTOR HDA. The agar plates were incubated for 1–2 days at 30°C and served as master plates from which different selection plates for confirmation of interactions were pinned. From every single positive clone from the screen, four technical replicates were pinned from SC-Leu-Trp plates onto agar selection plates with increasing stringency (SC-Leu-Trp-His, SC-Leu-Trp-His + 5 mM 3-AT, SC-Leu-Trp-His + 7.5 mM 3-AT or SC-Leu-Trp-His-Ade). Based on growth on selection plates, positive clones were selected, and cells were grown in 5 ml SC-Leu overnight at 30°C at a rotary shaker to lose the bait plasmid. Prey plasmids were isolated from yeast cells. To achieve maximum yield of prey plasmid recovery, *E. coli* DH5α cells were transformed with 10 μl of the plasmid mixture, plasmids were re-isolated and sequenced.

### Toxicity-Rescue Screen

The second high-throughput screening approach employed direct isolation of modifiers of αSyn toxicity by co-expressing αSyn gene without a tag along with roughly one million peptides from the PEP1170^+^ library. Yeast strain AH109 was transformed with plasmid pME3597, harboring αSyn encoding gene under *GAL1* promoter. Isolated single colonies were tested for growth by spotting assay. A clone with moderate growth inhibition upon expression of αSyn was mated with the PEP1170+ library as described in the Y2H screen procedure. After successful mating, the diploid cells were diluted and plated on 30 large SC-Leu-Trp agar plates (140 mm diameter), supplemented with 2% galactose for induction of αSyn expression. The plates were incubated at 30°C and observed for growth regularly. Colonies that appeared first were streaked on SC-Leu-Trp agar plates, supplemented with 2% glucose. Single colonies were used for prey plasmid rescue as described above and sequenced.

### Fluorescence Microscopy and Quantifications

Yeast cells were grown in SC selective medium containing 2% raffinose at 30°C overnight and transferred to 2% galactose containing medium for induction of αSyn-GFP expression for 6 h. Fluorescent images were obtained with Zeiss Observer. Z1 microscope (Zeiss) equipped with a CSU-X1 A1 confocal scanner unit (YOKOGAWA), QuantEM:512SC digital camera (Photometrics), and SlideBook 6.0 software package (Intelligent Imaging Innovations). For quantification of aggregation, at least 200 cells were counted per strain and per experiment. The number of cells presenting inclusions was referred to the total number of cells counted. The values are the mean of at least four independent experiments.

### Flow Cytometry

Cells were grown and protein expression was induced as described above. Yeast cell membrane integrity was analyzed with propidium iodide (PI) staining. Yeast cells were incubated with 12.5 μg/ml PI for 30 min in dark. Dihydrorhodamine 123 (DHR123) was used as an indicator for intracellular ROS accumulation. Yeast cells, expressing *GAL1*-driven αSyn-mCherry were incubated with DHR123 (Sigma-Aldrich) at a final concentration of 5 μg/ml for 1.5 h at 30°C. Before flow cytometry measurements, the cells were washed and re-suspended in 50 mM trisodium citrate buffer, pH 7.0. Flow cytometry analysis was performed on a BD FACSCANTO II (Becton Dickinson). Twenty thousand events were counted for each experiment. Data analysis was performed using the BD FACSDIVA software (Becton Dickinson).

### Immunoblotting

Yeast cells harboring αSyn-GFP were pre-grown at 30°C in SC selective medium containing 2% raffinose. Cells were transferred to SC medium containing 2% galactose at OD_600_ = 0.1 to induce the *GAL1* promoter for 6 h. Total protein extracts were prepared, and the protein concentrations were determined with a Bradford assay. Forty microgram of each protein were subjected to 12% SDS-polyacrylamide gel electrophoresis and transferred to a nitrocellulose membrane. Membranes were probed with anti-αSyn rabbit antibody (Santa Cruz, USA). GAPDH mouse monoclonal antibody (Thermo Fisher Scientific, USA) was used as a loading control. The use of actin as a loading control was avoided since yeast actin intron contains a cryptic promoter that is normally inactive, however, its deletion can lead to transcriptional interference (Irniger et al., 1992). Pixel density values for Western quantification were obtained from TIFF files generated from digitized X-ray films (KODAK) and analyzed with the ImageJ software (NIH, Bethesda, MD, USA).

### Human Recombinant αSyn Expression and Purification

The expression and purification of αSyn were performed as previously described (Miranda et al., 2013). Briefly, *E. coli* BL21 (DE3) cells were transformed with pET22b-αSyn construct, and expression of 500 ml LB culture was induced with isopropyl β-D-1-thiogalactopyranoside (IPTG) with the final concentration of 1 mM at OD = 0.3 at 37°C overnight. Cells were pelleted, frozen at −80°C, and re-suspended in lysis buffer (750 mM NaCl, 10 mM Tris pH 8.0, 1 mM EDTA, 1 mM protease inhibitor mix). Cells were lysed by sonication on ice (five times, 30 s each step, cool down for 1 min after each sonication step), heated at 95°C for 15 min, and then centrifuged at 13,000 rpm at 4°C for 20 min. The supernatant was dialyzed overnight at 4°C against dialysis buffer (50 mM NaCl, 10 mM Tris pH 7.6, 1 mM EDTA). αSyn was purified on two HiTrap Q FF 1 ml anion exchange columns (GE Healthcare) in 25 mM Tris pH 7.7 with a NaCl gradient from 0–600 mM. αSyn fractions were collected as judged by 12% SDS-polyacrylamide gel electrophoresis. αSyn was further purified by size-exclusion chromatography on a Superdex 75 26/600 prep grade 120 ml column (GE Healthcare) in SEC buffer (100 mM NaCl, 25 mM HEPES, 1 mM DTT, pH 8.0). The purification of αSyn was confirmed by SDS-PAGE and Western blotting analysis, and proteins were stored at −80°C.

### Expression and Purification of K50-His6

K50-His6 was found to be insoluble when expressed in *E. coli* BL21 (DE3), which is why hybrid conditions were used for the purification of the protein from bacterial cells. *E. coli* BL21 (DE3) cells were transformed with pET22b-K50-His6 construct, and expression of 100 ml LB culture was induced with isopropyl IPTG with a final concentration of 0.1 mM at OD = 0.2 at 30°C for 4 h. Cells were pelleted and re-suspended in 4 ml resuspension buffer (20 mM Tris pH 8.0). Cells were disrupted by sonication on ice (5 times, 30 s each step, cool down for 1 min after each sonication step), and then centrifuged at 13, 000 rpm in 4°C for 10 min. The supernatant was removed and the pellet was re-suspended in 3 ml cold isolation buffer (2 M urea, 20 mM Tris pH 8.0, 500 mM NaCl, 2% Triton-X 100). The sonication and centrifugation steps were repeated as above, the supernatant was collected and supplemented with imidazole to a final concentration of 10 mM. The protein was bound to Ni-NTA beads by agitation for 1.5 h at 4°C. The resin was settled by gravity and the beads were washed with 10 ml washing buffer (20 mM Tris pH 8.0, 10 mM Imidazole) and with 10 ml native washing buffer (50 mM Tris pH 8.0, 300 mM NaCl, 10 mM Imidazole). The protein was eluted with elution buffer (50 mM Tris pH 8.0, 300 mM NaCl, 250 mM Imidazole). The buffer was exchanged using Amicon^®^ Ultra-3K centrifugal filter device with 250 mM NaCl, 20 mM HEPES, pH 8.0.

### Ultracentrifugation and Fractionation

The sedimentation assay, extraction of SDS-soluble and insoluble αSyn protein fractions were performed as described (Popova et al., 2018). Equal amounts of yeast cells corresponding to total OD = 10 were collected by centrifugation and resuspended in lysis buffer (50 mM Tris-HCl pH 7.5, 1 mM EDTA, 5 mM DTT, 1× protease inhibitor mix (Roche). The cells were lysed by shaking with glass beads at 4°C. The crude lysate was centrifuged for 5 min at 500 g to pellet the cell debris. 200 μl of each cleared lysate was centrifuged at 100,000 *g* for 30 min. The supernatant was designated as a soluble protein. The pellet was washed 3 times with the lysis buffer, resuspended in 200 μl lysis buffer containing 2% SDS, and incubated on ice for 30 min. The suspension was centrifuged for 30 min at 100,000 *g* and the supernatant was labeled as SDS-soluble protein fraction. The pellet was washed three times with lysis buffer, resuspended in 200 μl 6 M urea, and designated as SDS-insoluble fraction (pellet). Equal amount from each fraction (20 μl) was analyzed by SDS-PAGE and Western blotting.

### Thioflavin T Assay

For the modified Thioflavin T (ThT) assay (Giehm and Otzen, 2010) αSyn was incubated at a final concentration of 30 μM or 50 μM in 25 mM HEPES (pH = 6.5), 100 mM NaCl, 0.002% SDS, and 100 μM Thioflavin T, either alone or in presence of the peptides at the indicated molar ratios in a final volume of 100 μl per well. The samples were incubated at 37°C for at least 100 h with continuous shaking in a black 96-well plate, pre-loaded with one glass bead per well, and covered with an adhesive plate sealer. Fibril formation was monitored by fluorescence every 2 min, with excitation at 440 nm and emission at 480 nm in a fluorescent plate reader (Tecan Infinite M200). Samples containing the peptides without αSyn were used as negative controls. All experiments were carried out in quadruplicates.

### Transmission Electron Microscopy (TEM)

αSyn aggregation products were analyzed by negative staining and transmission electron microscopy according to Hoppert and Holzenburg (Hoppert and Holzenburg, 1998). In brief, a carbon film, evaporated onto a freshly cleaved mica surface, was partially floated off on the surface of a sample droplet for 1 min. The carbon/mica sandwich was removed, allowing the carbon film to fall back into its original position. For washing, the sandwich was transferred to a water drop and treated in the same way for 3 s to remove buffer constituents. Staining of the sample was performed by slowly immersing the sandwich into phosphotungstic acid (pH 7.0, 3%); the carbon film was completely floated off. Electron microscope grids (300 mesh, Plano, Wetzlar, Germany) were used to pick up the floating carbon film. After the grid was removed from the droplet, excess fluid was blotted by touching the grid vertically with a piece of filter paper. After drying, the negative stained samples were imaged using a Jeol EM 1011 transmission electron microscope (Jeol, Eching, Germany), equipped with a Gatan Orius 4 K camera (Gatan, Munich, Germany).

### Human Cells

Human neuroglioma cells (H4) were maintained in Opti-MEM I Reduced Serum Medium (Life Technologies-Gibco) supplemented with 10% fetal bovine serum Gold (FBS) (PAA, Cölbe, Germany) at 37°C in an atmosphere of 5% CO_2_. The cells were plated in 12-well plates (Costar, Corning, New York) 24 h before transfection. H4 cells were transfected with FuGENE^®^ six Transfection Reagent (Promega, Madison, WI) according to the manufacturer’s instructions with equal amounts of plasmids of SynT and synphilin-1 as previously described (McLean et al., 2001; Lázaro et al., 2014). Twenty-four hours after the transfections, the cells were treated with K84s or K102s peptides at a concentration of 1 μM. 2% Ethanol was used as vehicle control. After 24 h, the cells were subjected to immunocytochemistry to examine αSyn inclusion formation.

### Immunocytochemistry

Twenty-four hours after exposition to the peptides, cells were washed with PBS and fixed with 4% paraformaldehyde for 10 min at room temperature. Cells were then permeabilized with 0.5% Triton X-100 (Sigma) for 20 min at room temperature and blocked in 1.5% normal goat serum (PAA)/PBS for 1 h. Cells were incubated overnight with mouse anti-αSyn primary antibody (1:1,000, BD Transduction Laboratories, NJ), and afterward with a secondary antibody (Alexa Fluor 568 donkey anti-mouse IgG) for 2 h at room temperature. Finally, cells were stained with DAPI (Life Technologies-Invitrogen) (1:5,000 in PBS) for 10 min and maintained in PBS for epifluorescence microscopy.

### Quantification of αSyn Inclusions

Transfected cells were scored based on the pattern of αSyn inclusions and classified as presented. Results were expressed as the percentage of the total number of transfected cells, and a minimum of 50 cells was counted *per* condition.

### Synthetic Peptides

Custom synthesis and analysis of the FITC-K84s and FITC-K102s was performed by the company ProteoGenix (Schiltigheim, France) (Supplementary Figure 1). Ten microliters of sample K102 was injected on an HPLC column (Kromasil 100-5C18, 4.6 × 250 mm, 5 μm) using a linear acetonitrile/0.1% (v/v) formic acid in H_2_O/0.1% (v/v) formic acid gradient (from 32% to 57% (v/v) acetonitrile/0.1 formic acid in 25 min, plus additional 5 min with 100% (v/v) acetonitrile/0.1 formic acid). 10 μl of sample K84 was injected on a HPLC column (Agela 100-5C18, 4.6 × 250 mm, 5 μm) using a linear acetonitrile/0.1% (v/v) formic acid in H_2_O/0.1% (v/v) formic acid gradient (from 35% to 60% (v/v) acetonitrile/0.1 formic acid in 25 min, plus additional 5 min with 100% (v/v) acetonitrile/0.1 formic acid). The flow rate was 1 ml/min. Detection was performed at 220 nm.

Peptides K50s, K89s, K94s, and K97s were synthesized by the company GenScript (Leiden, Netherlands) and analyzed at the Service Unit Metabolomics at the Institute of Microbiology and Genetics (University of Göttingen) (Supplementary Figure 2). Peptides were dissolved in AcCN/H_2_O (2% v/v) and analyzed with a Q Exactive™ Focus orbitrap mass spectrometer coupled to an UltiMate™ 3000 HPLC (Thermo Fisher Scientific). The pure solvent was used as control. Five microliters of each sample was injected on a HPLC column (Acclaim™ 120, C18, 5 μm, 120 Å, 4.6 × 100 mm (Thermo Fisher Scientific)) using a linear acetonitrile/0.1% (v/v) formic acid in H_2_O/0.1% (v/v) formic acid gradient (from 5% to 95% (v/v) acetonitrile/0.1 formic acid in 20 min, plus additional 10 min with 95% (v/v) acetonitrile/0.1 formic acid) with a flow rate of 0.8 ml/min at 30°C. The measurements were performed in a mass range of 70–1,050 m/z in positive mode with electrospray ionization (ESI). A Corona™ Veo™ RS Charged aerosol detector (CAD) (Thermo Fisher Scientific) was used as an additional detector. Data acquisition and analysis were performed with Thermo Scientific Xcalibur™ 4.1 (Thermo Fisher Scientific) and with FreeStyle™ 1.6 (Thermo Fisher Scientific) software.

## Results

### Screening for αSyn Interacting Peptides and for Suppressors of Toxicity

A peptide library consisting of approximately one million peptide variants was utilized for intracellular screening for interaction partners of αSyn using the yeast-two-hybrid (Y2H) method. The peptide library was generated by random insertion of 60 nt random sequences flanked by *Eco*RI and *Xho*I restriction sites into the scaffold B1 domain (nt 1483 - nt 1857) of protein G from *Staphylococcus aureus* at a site of a loop between two β-sheets (Fritz and Green, 1996). The random sequence may contain stop codons leading to peptides of variable length lacking the C-terminus of the protein GB1 domain. The library may also contain clones with a 61 nt insert that are not in frame with the C-terminal part of the protein GB1 domain resulting in a C-terminal addition of the sequence PSRH* to the random peptide. The small peptides are expressed as a fusion to the B42 transcription activation domain (AD) (“prey”). The “bait” protein αSyn was expressed as a fusion to the *GAL4* DNA-binding domain (BD). The Y2H screen was performed by selection for cellular yeast growth as previously described (James et al., 1996). Productive interaction between the bait and the prey brings the transcription activation and DNA-binding domains together and initiates the expression of two biosynthetic reporter genes (*ADE2* and *HIS3*) that allow growth in the absence of histidine and adenine (Supplementary Figure 3). No background yeast growth was observed in the Y2H screening indicating that the GB1 scaffold that is present in all clones is not able to either interact with the bait protein or alter its toxic properties. An optimized interaction-mating protocol was used for improved yield of double transformants to cover efficiently the complexity of the peptide library (Soellick and Uhrig, 2001). Optimization of screening conditions was performed by testing for auto-activation of the bait construct (αSyn) aiming to increase the stringency and avoid false positives. Two rounds of Y2H screenings were independently performed to achieve unbiased results and to increase the chances for the identification of interaction partners. Two-hundred and thirty-four clones were selected that grew on a prototrophic semi-solid growth medium without histidine. These putative interaction partners from the initial Y2H screen were validated for their potential to induce the expression of the reporter genes on selective media in a secondary screen. The clones were inoculated in a liquid selective medium, and 90 clones were selected that showed the most pronounced growth for further validation. Growth assays were performed in an ordered array using an automatic robotic platform on multiple selection plates with increasing stringency, achieved by the addition of different concentration of the histidine analogue 3-AT (3-Amino-1,2,4-triazole), a competitive inhibitor of the enzyme derived of the *HIS3* gene, and double selection in the absence of histidine and adenine (Supplementary Figure 4A). Thirty-seven clones were selected based on their growth on selection media, plasmids were rescued and sequenced.

A second alternative and novel screening approach was used to directly select peptides that suppress the toxicity of αSyn (“toxicity-rescue screen”). A constructed yeast strain with moderate growth inhibition upon expression of αSyn (Supplementary Figure 4B) was mated with yeasts of the opposite mating type carrying the peptide library. Screening for yeast diploid cells that revealed enhanced growth upon expression of individual library variants was performed on large selection plates, supplemented with galactose for induction of αSyn expression. Colonies that appeared during the first 3 days of growth at 30°C were picked and the corresponding plasmids were isolated and amplified in bacteria. Thirty-one unique individual library representatives were characterized by sequencing.

### Seven Peptide Constructs Suppress αSyn Toxicity and Aggregation

**Figure 1:**
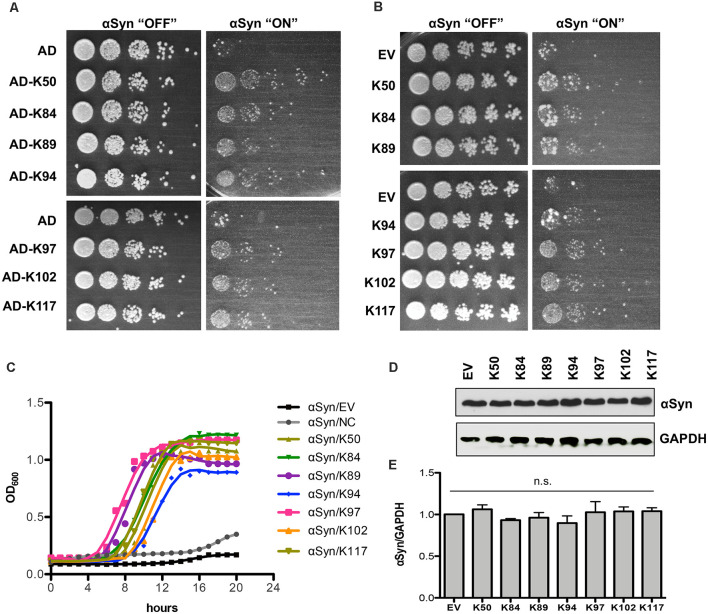
Expression of seven peptide constructs reduces αSyn-induced growth inhibition. **(A)** The yeast strain that harbors three copies of *GAL1*-driven αSyn-GFP-encoding gene (inducible “high-tox” strain RH3468) was transformed with the plasmid constructs, isolated from the screen. The Y2H plasmids contain the peptide-encoding sequence, fused to the B42-activation domain (AD). Yeast cells were spotted in 10-fold dilution on SC-Leu-Ura selective plates containing glucose (control: αSyn “OFF”) or galactose (αSyn “ON”) for induction of *GAL1* promoter. Plasmid with AD was used as a control. Images of the plates were captured on the third day. **(B)** The peptide-encoding sequences were re-cloned without the N-terminal activation domain and driven by the *GPD* promoter. The yeast high-tox strain was transformed with the corresponding plasmids and spotting assays were performed. Empty vector (EV) was used as a control. **(C)** Growth analyses of yeast cells from **(B)** in galactose-containing medium for 20 h. **(D)** Immunoblotting analysis of protein crude extracts of yeast cells from **(B)** after 6 h induction in galactose-containing medium with an anti-αSyn antibody. Anti-GAPDH antibody was used as a loading control. **(E)** Densitometric analysis of the immunodetection of αSyn-GFP, relative to the loading control GAPDH. n.s.: not significant, (*n* = 3).

The isolated peptide constructs from the Y2H screen and toxicity-rescue screen were individually tested for their ability to suppress αSyn toxicity in yeast. The starting point was an inducible “high-tox” yeast strain that harbors three genomically integrated αSyn-GFP-encoding gene copies resulting in a strong growth retardation phenotype (Petroi et al., 2012), which was transformed individually with the peptide constructs. Spotting tests were performed to determine the effect of different peptide constructs on yeast growth. Seven clones demonstrated reproducible suppression of αSyn toxicity, as shown by the spotting test (Figure 1A). Two of them (K50 and K117) were isolated from the Y2H screen and five peptide constructs (K84, K89, K94, K97, K102) were isolated from the toxicity-rescue screen. There were no identical peptides, isolated from both screens. The toxicity suppression effect was specific for the isolated peptides since growth tests of yeast cells carrying random peptides from the screen as a control revealed no growth enhancement effect (Supplementary Figure 5A). These results suggest that the modulation of αSyn toxicity is attributed to the unique peptide sequences. Next, the peptide-encoding sequences including the flanking scaffold protein sequences but without the bulky activation domain were re-cloned behind the constitutive *GPD* promoter (Table 3). K50 and K117 contain the full-length scaffold protein sequence plus the variable part, whereas the peptides isolated from the toxicity-rescue screen had premature stop codons and were shorter in length. Yeast high-tox strain was re-transformed with the new constructs. Growth assays on solid and in liquid medium revealed similar suppression of αSyn-induced toxicity when the peptides were expressed without the N-terminal activation domain (Figures 1B,C). Expression of the peptide constructs alone did not affect the growth of yeast wild-type cells (Supplementary Figure 5B). Immunoblotting analysis revealed similar steady-state levels of αSyn protein in the presence and absence of the peptide constructs after 6 h induction of protein expression (Figures 1D,E) indicating that the differences in toxicity are not due to changed αSyn protein levels.

**Table 3:** Amino acid sequences of peptides expressed in yeast.

Peptide construct	Amino acid sequence
K50	MYKLILNGKEFSPRWARTVWRASMGALAIMLALETLKGETTTEAVDAATAEKVFKQYANDNGVDGEWTYDDATKTFTVTE
K84	MYKLILNGKEFLVWGCLRGSAIGECVVHGGPPSRH
K89	MYKLILNGKEFVQGLMPRRAAWGGRSSRGRWPSRH
K94	MYKLILNGKEFIARSMGNMRMSERRRG
K97	MYKLILNGKEFLGCLPLSTAPLACWRTG
K102	MYKLILNGKEFLKRWARSTRWGTASCGGS
K117	MYKLILNGKEFGMILRMCWRVAQMMRVPLCLALETLKGETTTEAVDAATAEKVFKQYANDNGVDGEWTYDDATKTFTVTE

*The sequence of the GB1-scaffold protein is highlighted in red. K50 and K117 were isolated from the Y2H screen. The other peptides were isolated from the toxicity-rescue screen*.

**Figure 2:**
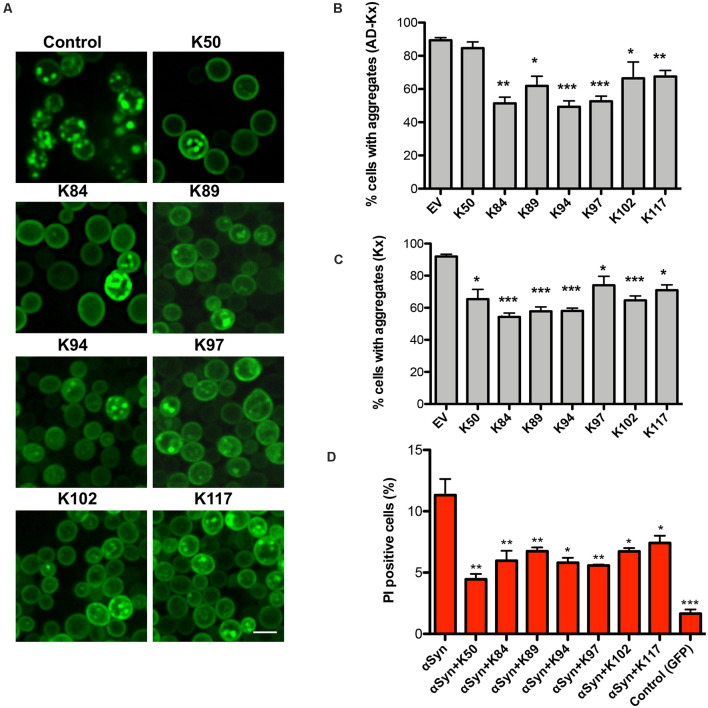
Peptide-mediated reduction of αSyn-GFP aggregation and increased cell viability. **(A)** Life-cell microscopy 6 h after induction of *GAL1*-αSyn-GFP expression in galactose-containing medium. Yeast cells (RH3468) were co-transformed with plasmids harboring the indicated peptide constructs under the constitutive *GPD* promoter or empty vector as a control. Scale bar: 2 μm. Quantification of the percentage of cells with αSyn aggregates, co-expressing AD-peptide (AD-Kx) **(B)** or the peptides without AD (Kx) **(C)**. Significance of differences was calculated with *t*-test (*p < 0.05; **p < 0.01; ***p < 0.001; *n* = 4). **(D)** Propidium iodide (PI) fluorescence intensity of yeast cells, expressing αSyn and indicated peptides after 24 h of induction was assessed by flow cytometry. Quantification of PI-positive cells with higher fluorescent intensities than the background 6 h after induction of αSyn expression. Cells expressing GFP were used as a negative control. Significance of differences was calculated with *t*-test relative to αSyn (*p < 0.05; **p < 0.01; *n* = 4).

We analyzed whether the expression of the peptides affects the aggregate formation of αSyn-GFP. In yeast, the number of cells with aggregates is an established and very sensitive variable that correlates with cytotoxicity (Outeiro and Lindquist, 2003; Petroi et al., 2012). Fluorescent microscopy studies were performed with αSyn high-tox strain in the presence and absence of peptide constructs (Figure 2A). The numbers of cells displaying aggregates were significantly reduced upon co-expression of AD-peptides or peptides without the AD, in comparison to the control (Figures 2B,C). Expression of the peptides might affect also other toxic forms of αSyn that precede aggregation.

To assess, whether the differences in growth and aggregate formation correlate with changes in cytotoxicity, propidium iodide (PI) staining for membrane permeability was performed as a sensitive method for quantification of yeast viability. Flow cytometry measurements of cells, expressing αSyn and the peptide constructs showed a significantly decreased number of PI-positive cells in comparison to αSyn expression in the absence of peptide expression (Figure 2D). These results correlate with the data from the growth assays and reveal that the expression of the seven peptides significantly reduces αSyn aggregate formation and enhances cell growth and viability.

### Peptides Reduce the Accumulation of Reactive Oxygen Species

**Figure 3:**
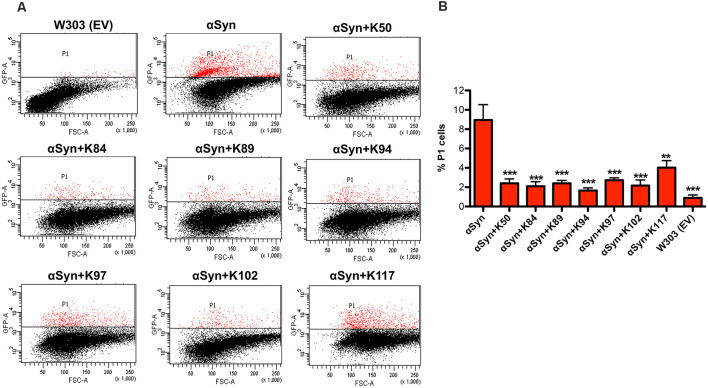
Peptide expression reduces the number of yeast cells that accumulate αSyn-induced reactive oxygen species (ROS). **(A)** αSyn expression was induced for 6 h in the galactose-containing medium. Cells were incubated with dihydrorhodamine 123 (DHR123) as an indicator of intracellular ROS accumulation for 1.5 h and analyzed by flow cytometry. Dot plots of side scatter and DHR123-fluorescence for wild type yeast (W303) as a negative control, expression of αSyn alone and αSyn in presence of the indicated peptides. P1: subpopulation of yeast cells with higher fluorescent intensity. **(B)** Quantification of the number of cells with higher fluorescent intensity (P1). The significance of differences was calculated with *t*-test vs. αSyn (**p < 0.01; ***p < 0.001; *n* = 3).

Oxidative stress is a central event in PD that triggers αSyn misfolding and aggregation (Giasson et al., 2000). We analyzed the effect of peptide expression on the accumulation of reactive oxygen species (ROS) by using the dye Dihydrorhodamine 123 (DHR123). DHR123 accumulates in cells, where it is oxidized by free radicals to the bright green fluorescent product rhodamine 123. Expression of αSyn resulted in a significant increase in the number of cells accumulating ROS in comparison to the wild-type cells, as assessed by flow cytometry. Co-expression of the peptides significantly reduced the number of cells that accumulate ROS (Figure 3). These results indicate that the expression of the seven peptides affects specific molecular pathways that attenuate the accumulation of ROS.

### The Peptide-Mediated Suppression of Toxicity Is Specific for αSyn

**Figure 4:**
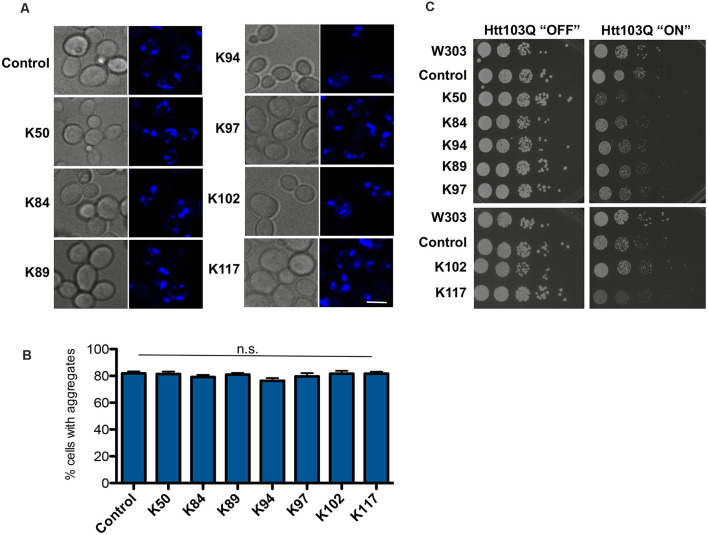
Huntingtin-103Q aggregation and cellular yeast growth phenotypes are independent of the presence of αSyn-specific peptides. **(A)** Life cell microscopy of yeast strain RH3788, co-expressing huntingtin construct with 103 PolyQ repeats (Htt103QΔPro-CFP) and the indicated peptide constructs or empty vector as a control. Yeast cells, pre-grown to mid-log phase, were induced in galactose-containing medium and examined for aggregates at 6 h of induction. Expression of the Htt103Q variant revealed the formation of fluorescent foci. Scale bar: 5 μm. **(B)** Aggregate quantification of yeast cells, expressing Htt103Q construct. For each strain, the number of cells displaying cytoplasmic foci is presented as a percent of the total number of cells counted. For quantification of aggregation at least 200 cells were counted. **(C)** Yeast cells from **(A)** were spotted in 10-fold dilutions on selection plates containing glucose (Htt103Q “OFF”) or galactose (Htt103Q “ON”) for induction of *GAL1* promoter. W303—wildtype yeast background strain, transformed with empty vectors. Control: Htt103QΔProCFP strain, transformed with empty vector. n.s.: not significant.

The specificity of the effect of peptide expression for αSyn was evaluated. The seven peptide constructs were transformed into a yeast disease model of Huntington disease that expresses a toxic polypeptide unrelated to αSyn. Expression of exon one of human huntingtin with 103 glutamine residues (Htt103Q) is toxic to yeast cells and forms aggregates (Duennwald et al., 2006). A yeast strain that expressed the Htt103Q-CFP construct with modest toxicity was constructed. Expression of the peptide constructs did neither suppress toxicity nor aggregate formation (Figure 4). These results indicate that the observed effects of peptide construct expression are specific to αSyn and that there is no indication of overlap with the molecular mechanisms, suppressing the Htt103Q toxicity. Interestingly, expression of K50 and K117 increased the Htt103Q toxicity, indicating distinct pathways involved in general protection from misfolded proteins in yeast. These results support previous findings from genetic screens in yeast that genes suppressing the toxicity of αSyn and Htt103Q do not overlap (Willingham et al., 2003; Cooper et al., 2006).

### Expression of K50, K84, and K102 Reduces the Levels of Insoluble αSyn

**Figure 5:**
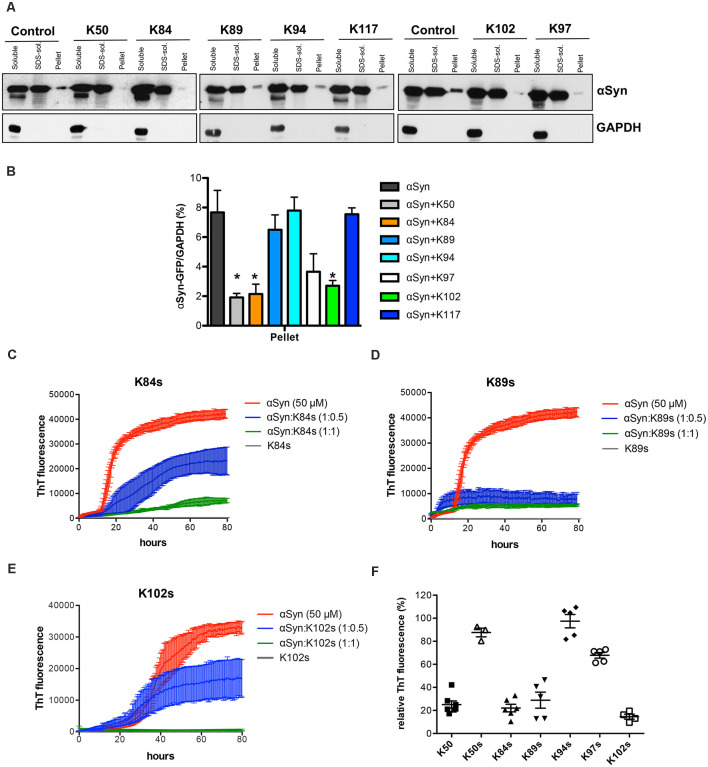
Distribution of αSyn-GFP levels across different solubility fractions. **(A)** Yeast cells, expressing *GAL1*-driven αSyn-GFP from three genomic copies and the indicated peptide constructs or the corresponding empty vector (control) were induced for 6 h in galactose medium. Starting from an equal number of cells, crude protein extracts were prepared and fractionated by ultracentrifugation to produce soluble, SDS-soluble and SDS-insoluble fractions (pellet), revealing the level of insoluble αSyn. Equal amounts from all fractions were analyzed by immunoblotting with anti-αSyn. The membranes were stripped and probed with an anti-GAPDH antibody as a loading control. **(B)** Densitometric analysis of the immunodetection of αSyn-GFP relative to GAPDH loading control. The significance of differences was calculated with *t*-test vs. αSyn (*p < 0.05; *n* = 4). **(C)** Peptide-mediated effect on the amyloidogenic properties of αSyn *in vitro*. Aggregation kinetics of αSyn in the presence and absence of the synthetic peptides K84s **(C)**, K89s **(D)**, or K102s **(E)** monitored by ThT fluorescence emission. αSyn was incubated at a concentration of 50 μM in the absence or upon addition of the peptides in the indicated molar ratios at time point 0. The ThT fluorescence emission was recorded every 15 min for 100 h. Incubation of the peptides alone served as a control. Representative curves showing the fluorescent signal over time. **(F)** Quantification of the relative ThT fluorescence signal in presence of the indicated peptides at molar ratio 1:1 (1:0.2 for K50) after 100 h relative to αSyn alone. The data are mean of five independent experiments.

The solubility of αSyn in cells expressing different peptide constructs was determined to characterize the effect of peptide construct expression on the biochemical nature of αSyn species in yeast cells. Six hours after induction of protein expression crude protein extracts from equal numbers of cells were prepared. The protein extracts were subjected to fractionation to produce soluble, SDS-soluble, and SDS-insoluble fractions, and equal amounts of the fractions were loaded on SDS gel (Figure 5A). Comparison of the different fractions revealed significant decreases of SDS-insoluble αSyn (pellet) in cells, expressing K50, K84, and K102 compared to controls (Figure 5B). This suggests that K50, K84, and K102 inhibit insoluble αSyn species formation in living cells.

### K84s and K102s Peptides Inhibit αSyn Aggregation *In vitro*

The formation of αSyn amyloid fibrils can be reproduced *in vitro* by incubation of recombinant αSyn protein in the presence of Thioflavin-T (ThT), a dye that specifically binds to amyloid fibrils. We compared the aggregation properties of αSyn in the absence and presence of each of the identified synthetic peptides to determine, whether the peptides directly affect the kinetics of αSyn amyloid formation. Minimal peptide sequences shortened to the variable region were deduced based on secondary structure predictions of the peptides that were expressed in yeast (Table 4). Flanking amino acids were incorporated where they stabilized the secondary structure as predicted with PEP FOLD 3 (Shen et al., 2014) and the peptides were chemically synthesized (denoted as **Ks**; Supplementary Figure 6). Among the seven isolated peptides, K50 and K117 contain the C-terminal scaffold domain and are longer. K50 was expressed in *E. coli* and purified as a His-tagged recombinant protein. Attempts to purify soluble K117 in *E. coli* were unsuccessful, hence the peptide was not further used for *in vitro* studies. αSyn recombinant protein was purified as described (Miranda et al., 2013). Fifty micromolar αSyn was incubated with the peptides at molar ratios of 1:0.5 or 1:1, added at time point 0. The kinetics of aggregation was monitored by ThT fluorescence every 15 min for 100 h. The addition of peptides K50 at a molar ratio of 1:0.2 and K84s, K89s, and K102s at a molar ratio of 1:1 reduced the ThT signal by more than 75%, indicating that the peptides were able to inhibit the aggregation of αSyn (Figures 5C–F, Supplementary Figure 7). At increasingly stoichiometric ratios, we observed progressively reduced inhibition consistent with a general dose-dependence. A modest reduction of αSyn aggregation was observed for K97s (Supplementary Figure 7), whereas addition of K50s and K94s had no effect on αSyn aggregation (Supplementary Figure 7). K89s and K102s reduced the lag phase of the aggregation process, while at the same time reducing the maximum ThT fluorescence signal, indicating a reduction in aggregation. These data suggest that the peptides change the fibrilization kinetics and the overall formation of ThT-positive species.

**Table 4:** Amino acid sequences and physicochemical properties of chemically synthesized short peptides.

Synthetic peptide	Amino acid sequence	Length (aa)	Charge	Nature	pI
K50s	FSPRWARTVWRASMGALAIMLALE	24	2	basic	12.2
K84s	FLVWGCLRGSAIGECVVHGGPPSRH	25	3	basic	8.2
K89s	VQGLMPRRAAWGGRSSRGRWPSRH	24	7	basic	13.2
K94s	IARSMGNMRMSERRRG	16	4	basic	12.7
K97s	LGCLPLSTAPLACWRTG	17	1	basic	8.2
K102s	FLKRWARSTRWGTASCGGS	19	4	basic	12.2

**Figure 6:**
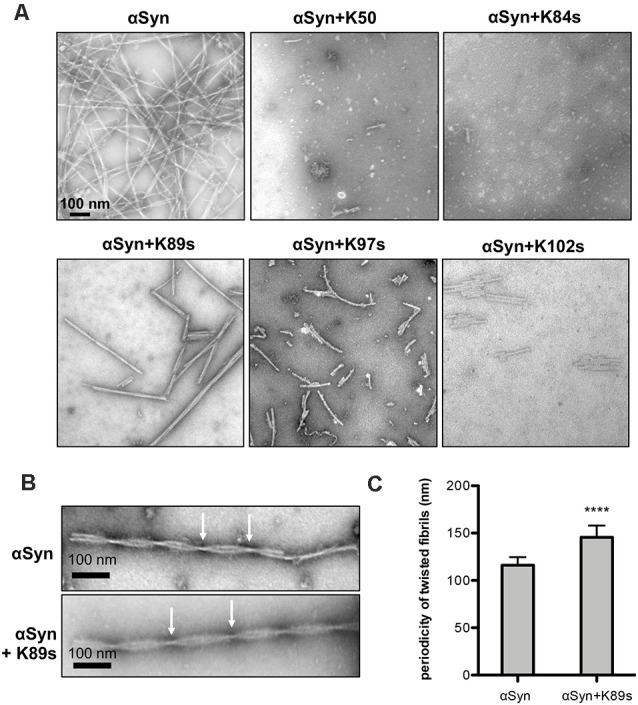
Fibril morphology of αSyn in absence and presence of peptides. **(A)** Representative transmission electron microscopy (TEM) images showing endpoint samples of *in vitro* formed αSyn fibrils after 100 h. **(B)** Periodicity of the fibrils, formed by control αSyn sample and in presence of K89s. Arrows mark crossovers of two twisting protofibrils. **(C)** Quantification of the mean fibril periodicity of αSyn in the absence and presence of K89s. The significance of differences was calculated with a *t*-test (****p < 0.0001; *n* = 50).

As a second direct qualitative measure for fibril formation, samples used in continuous growth experiments were imaged using transmission electron microscopy (TEM). We analyzed the morphological features of the aggregates at a stoichiometry of 1:1 (1:0.2 for K50) since it was most effective in ThT experiments. αSyn control formed typical long unbranched amyloid fibrils (the fully assembled polymer), formed by two twisted protofibrils (Figures 6A,B), similar to observed structures of αSyn fibrils (Dearborn et al., 2016; Guerrero-Ferreira et al., 2018). Samples incubated with K50, K84s and K102s were inhibited in fibril formation. Small round globular structures that resemble in morphology observed oligomeric species (Cappai et al., 2005; Pieri et al., 2016), and a few short fibrils could be observed. Aggregation in presence of K89s resulted in the formation of long twisted fibrils. We measured the periodicity of αSyn fibrils formed in the presence and absence of K89s. Based on measurements of straight fibrils outside of crowded regions, the periodicity was found to be significantly increased from 116 ± 8.5 nm for αSyn to 145.5 ± 12.5 nm in presence of K89s (Figures 6B,C). Thus, K89s changed the fibril morphology that might translate into differences in cytotoxicity. Similarly, the C-terminally truncated form of αSyn that shows stronger cytotoxicity (Tanaka et al., 2019) forms twisted fibrils with reduced periodicity (Iyer et al., 2017). The addition of K97s reduced the fibril load and the length of the fibrils. These data corroborate the observed effects of peptides in living cells and indicate direct interference of the peptides with αSyn amyloid formation. The results suggest K84s and K102s synthetic peptides as the most promising inhibitors of αSyn aggregation due to their short length and inhibition propensity.

### K50, K84s, K89s, and K102s Act at Early Steps of Aggregation by Modulating the Oligomeric State of αSyn

**Figure 7:**
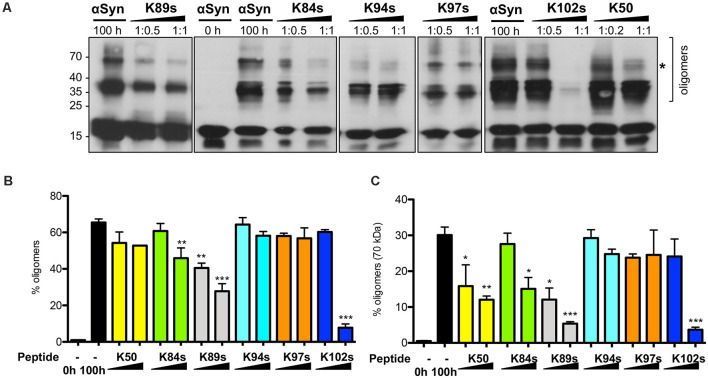
Peptide-mediated reduction in αSyn oligomerization *in vitro*. **(A)** αSyn was incubated in the absence or presence of peptides in the indicated molar ratios. A sample at the initial time point (0 h) served as a control. Equal amounts of samples were collected after 100 h and analyzed by immunoblotting with the anti-αSyn antibody. **(B)** Densitometric analysis of the immunodetection of αSyn oligomeric species. The relative amount of αSyn oligomeric species was normalized to the total immunodetection/well. **(C)** The relative amount of higher molecular weight αSyn oligomeric species (70 kDa band, indicated with *), normalized to the total immunodetection/well. Significance of differences was calculated with *t*-test vs. αSyn without addition of peptides (black bars) (*p < 0.05; **p < 0.01; ***p < 0.001; *n* = 3).

We investigated whether the inhibition of αSyn aggregation by the peptides is accompanied by modulation of the low molecular weight oligomeric species. Equal amounts of samples were collected from the endpoint of aggregation reactions and analyzed by SDS-PAGE and immunoblotting (Figure 7). αSyn SDS-stable oligomeric species, migrating at 15 kDa, 36 kDa, and 70 kDa were readily detected, corresponding to monomeric and different oligomeric αSyn species. The band intensity of the oligomeric species was evaluated by densitometry and compared to the control, where no peptide was present. A decrease in the accumulation of αSyn oligomeric species was observed with increasing concentration of K84s, K89s, and K50. The addition of K102s almost completely abolished the formation of αSyn oligomers. These results suggest that K50, K84s, K89s significantly suppress the first steps of oligomerization without completely inhibiting it, whereas K102s strongly inhibit the formation of low molecular weight αSyn oligomers.

### K84s Reduces the Formation of αSyn Aggregates in Human Cells

**Figure 8:**
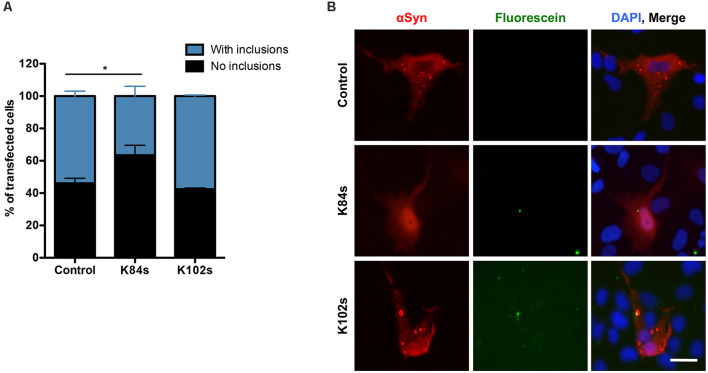
K84s reduces αSyn aggregation in human cells. **(A)** H4 cells were transfected with αSyn and treated with K84s (1 μM) or K102s (1 μM). Control: 2% ethanol (vehicle). Significance of differences was calculated with one-side *t*-test (*p < 0.05; *n* = 3). **(B)** Representative images of transfected cells treated with 1 μM of K84s and K102s. Scale bar = 30 μm.

Finally, we tested the ability of K84s and K102s to modulate αSyn aggregation in human cells. Human neuroglioma cells (H4) have been extensively used to model αSyn aggregation. In this model, cells co-expressing a C-terminally modified αSyn (SynT) and synphilin-1, exhibit the formation of αSyn inclusions, as previously described (McLean et al., 2001; Lázaro et al., 2014). We have previously shown that not all αSyn inclusions formed display amyloid-like properties and are Thioflavin positive, as they likely represent different types of protein aggregates (Lázaro et al., 2014). The peptides were labeled with fluorescein (FITC) to follow their internalization into the cells. However, due to the low amounts used in this study, especially for K102, the signal was low. Twenty-four hours after transfection, the cells were incubated with 1 μM K84s or K102s peptide. αSyn inclusion formation was assessed after 24 h. Treatment with 1 μM K84s resulted in a significant reduction in the percentage of cells with inclusions (Figure 8).

## Discussion

The aggregation of αSyn into amyloid fibrils is a major pathogenic process in PD and other synucleinopathies (Lashuel et al., 2013; Villar-Piqué et al., 2015). Accumulating evidence suggests that oligomers/protofibrils exert damaging effects in the affected cells. Membrane damage, mitochondrial defects, and synaptic dysfunction are some examples of the proposed pathogenic mechanisms (Bengoa-Vergniory et al., 2017). This suggests that inhibition of αSyn oligomerization and conversion into fibrillar aggregates is a viable strategy for therapeutic intervention in PD. The development of PD therapies that reduce or block αSyn aggregation is a priority goal in Parkinson’s research. Such therapies could potentially prevent or delay the onset of PD or slow its progression.

Peptides are very promising candidates for drug development, especially because of their small size, functional diversity, and a high degree of specificity towards a given target. Short peptides are composed of natural amino acids and their degradation is less likely to be toxic in comparison to synthetic small molecules. Moreover, they can be quickly and cheaply synthesized and also can undergo several chemical modifications for tuning of their properties such as membrane permeability or stability. Currently, there are more than 60 FDA-approved peptide drugs on the market, including peptide inhibitors for the treatment of neurodegenerative diseases (Fosgerau and Hoffmann, 2015; Baig et al., 2018). Most of the attempts to isolate peptides able to inhibit αSyn aggregation utilize semi-rationally designed libraries of peptides spanning parts of the N-terminal domain (1–60), which contains a multi-repeated consensus sequence (KTKEGV) and has an alpha-helical propensity, or the central domain (61–95), known as non-amyloid-beta component (NAC) that is highly hydrophobic and is involved in αSyn aggregation (El-Agnaf et al., 2004; Abe et al., 2007; Cheruvara et al., 2015; Torpey et al., 2020). Yeast was successfully used before only for the selection of candidates from a cyclic octamer peptide library that were able to rescue αSyn toxicity (Kritzer et al., 2009). In this study, we isolated short peptides that inhibit αSyn induced toxicity and aggregation using yeast as a screening platform. For the first time, a random library of short linear peptides was expressed in the PD model and directly screened for toxicity-rescue. This approach has the advantage that the candidates are selected based on the phenotype rather than affinity and toxic sequences are avoided.

We screened a library with a diversity ~1 × 10^6^ random amino acid sequences with a length of 20 residues, inserted into the B1 domain of protein G from *Staphylococcus aureus* at the site of a loop between two β-sheets as a scaffold. The diversity of the library represents only a small fraction of the formal sequencing space of 20^20^ possible sequences that are beyond a real-life experiment. However, protein interactions are governed frequently by just a few hydrophobic and/or charged residues spaced in the appropriate way that offers a large number of participating peptide sequences with properties suitable for interaction with a target protein. The scaffold stably folds into a 3D structure, which enhances the presentation of the random peptides towards the target protein. Seven peptides could be identified that can diminish αSyn aggregation and toxicity in yeast. Plasmid-borne expression of the peptides rescued the strong growth retardation phenotype of a high-tox yeast strain, expressing αSyn and increased cell viability. Importantly, the expression of the peptides significantly reduced the number of cells with aggregates. This effect was specific for αSyn since peptide expression had no impact on the aggregate formation of Htt103Q, a toxic and aggregation-prone polypeptide involved in Huntington’s disease.

Two consecutive optimization steps resulted in the identification of minimal peptide sequences that are still able to inhibit αSyn aggregation: (i) the peptides were expressed without the bulky N-terminal activation domain, keeping the first 11 amino acids from the scaffold protein; (ii) the peptide sequences of K50, K84, K89, K94, K97, and K102 were shortened to the variable region and synthetic peptides (denoted with **s**) with a length between 16 and 25 amino acids were used for *in vitro* aggregation assays. The addition of peptides K50, K84s, K89s, and K102s reduced αSyn amyloid formation *in vitro* by more than 75%. The ability of the peptides to inhibit αSyn aggregation was confirmed with TEM assays. The addition of K50 and K84s revealed the formation of oligomeric assemblies of αSyn similar to previously observed (Carija et al., 2019). The activity of the peptides was concentration-dependent and still evident in the sub-stoichiometric ratio of 1:0.5. This suggests that the peptides bind to oligomeric rather than to monomeric αSyn species. The synthetic K50s peptide corresponding to the short variable region of K50 lost the inhibition activity, suggesting changes in the structural properties of the polypeptide.

Oligomers are the intermediate species of the αSyn aggregation process. Their characterization is difficult due to their heterogeneity and variability. *in vitro*, both, on-pathways and off-pathways oligomers have been identified that exhibit different structural properties, suggesting multiple aggregation pathways (Villar-Piqué et al., 2015). Recently, Paslawski et al. reported the formation of two types of αSyn oligomers—one that can be elongated by monomers to form fibrils and a second one that stacks together to form more amorphous structures (Paslawski et al., 2014). The identification and expression of αSyn variants prone to oligomerization, but not fibrillization, suggests that oligomeric species might be the most toxic forms of αSyn (Karpinar et al., 2009; Winner et al., 2011; Rockenstein et al., 2014). Peptides K84s, K89s, and K102s significantly reduced the accumulation of soluble oligomeric species *in vitro*. This was accompanied by reduction of αSyn-induced toxicity by expression of the peptides in yeast cells, as well as reduction of the SDS-insoluble αSyn fraction by expression of K84 and K102 *in vivo*. These data propose K84s and K102s as potent suppressors of toxicity by reducing the load of oligomeric species and inhibiting the aggregation process of αSyn. This might have also important implications for the pathological spreading of the disease in the brain, since oligomeric species are proposed as candidates for mediating the prion-like spreading of αSyn aggregation (Luk et al., 2009, 2012; Hansen et al., 2011). Using fluorescently-labeled peptides, we demonstrated that they can be internalized into human cells. Importantly, peptide K84s reduced αSyn aggregation in human cells, suggesting it might constitute a promising new lead for the development of novel therapeutic strategies for synucleinopathies.

The peptides obtained from the screenings may serve as a starting point to dissect the amino acids involved in interaction and rescue function. One of the next important steps will be the mapping of the interaction sites of the peptides with αSyn for understanding the anti-aggregation mechanism. This may direct the synthesis of shorter peptides or peptidomimetic synthetic molecules that may be suitable as anti-αSyn-aggregation drugs.

## Data Availability Statement

The original contributions presented in the study are included in the article/Supplementary Material, further inquiries can be directed to the corresponding author.

## Author Contributions

Conceived and designed the experiments: BP and GB. Performed the experiments: BP, DW, AR, KD, DL, MH, and JG. Analyzed the data: BP, DW, AR, KD, DL, MH, TO, and GB. Contributed reagents/materials: JU. Wrote the article: BP and GB. All authors discussed and reviewed the manuscript. All authors contributed to the article and approved the submitted version.

## Conflict of Interest

The authors declare that the research was conducted in the absence of any commercial or financial relationships that could be construed as a potential conflict of interest.

## Supplementary Material

FSUPPLEMENTARY FIGURE 1 Analysis of peptides FITC-K84s and FITC-K102s. The upper panels represent the HPLC chromatograms of the samples. The lower panels represent the mass spectrometry spectrum of the sample peaks, marked with an asterisk in the upper panel.

SUPPLEMENTARY FIGURE 2 Analysis of peptides K50s, K89s, K94s, and K97s. The peptides were analyzed with LC-MS equipped with a charged aerosol detector (CAD). The upper panel shows the CAD chromatogram of the peptide sample (above) compared to the solvent control (below). Y-axis gives the relative current in %. The lower panel shows the MS spectrum of the peptide peak, marked with an asterisk in the upper panel.

SUPPLEMENTARY FIGURE 3 Flowchart depicting yeast-two-hybrid (Y2H) screening. Y2H assay is a step-by-step procedure starting from the construction of bait (αSyn), fused to the *GAL4* DNA-binding domain (BD) and amplification of the library, where each peptide construct is fused to B42 activation domain (AD). The bait is transformed into AH109 yeast strain that harbors *HIS3* and *ADE2* reporter genes driven by a *GAL4* regulatory binding site to select for growth on media lacking histidine and adenine and was mated with the library. Selection for diploids is performed on SC-Leu-Trp medium. Positive clones are selected by their ability to activate the transcription of the reporter genes that enable growth on SC-Leu-Trp-His medium.

SUPPLEMENTARY FIGURE 4 Exemplary growth assay for selection of library candidates. **(A)** Ninety colonies were picked from the initial selection on SC-Leu-Trp-His semi-solid medium after 6 days of incubation at 30°C. The cells were spotted on SC-Leu-Trp plate, allowed to grow for 2 days, and pinned in four technical replicates onto agar selection plates with increasing stringency. Red boxes indicate clones, selected for plasmid isolation and sequencing. **(B)** Spotting assay of yeast strain expressing *GAL1*-driven αSyn used for the toxicity-rescue screen. Yeast strain AH109 was transformed with an integrative vector harboring αSyn-encoding gene or empty vector (EV) as a control. Yeast cells were spotted in 10-fold dilution on selective SC-Trp plates, containing 2% glucose or galactose and allowed to grow for 3 days at 30°C.

SUPPLEMENTARY FIGURE 5 Expression of the peptide constructs does not affect yeast growth. **(A)** Expression of random peptide constructs does not rescue αSyn-induced growth inhibition. A yeast strain that harbors three copies of *the GAL1*-driven αSyn-GFP-encoding gene was transformed with three random plasmid constructs that contain the peptide-encoding sequence fused to the B42-activation domain (AD), or with a vector with AD. Yeast cells were spotted in 10-fold dilution on SC-Leu-Ura selective plates containing glucose (control: αSyn “OFF”) or galactose (αSyn “ON”) for induction of *GAL1* promoter. Yeast wild-type isogenic background strain transformed with AD-vector was used as positive growth control. **(B)** Yeast strain, expressing *GAL1*-driven GFP was transformed with the plasmids, encoding the peptide sequences without the N-terminal AD. Spotting assay was performed as described. Empty vector (EV) was used as a control.

SUPPLEMENTARY FIGURE 6 Secondary structure predictions of the indicated peptides. PEP FOLD 3 tool was used for predicting peptide structures from amino acid sequences. **(A)** Peptide constructs with N-terminal GB1-scaffold expressed in yeast. K50 and K117 contain also the C-terminal scaffold sequence, as indicated in Table 3. **(B)** Predicted structure of the synthetic peptides consisting only of variable region sequences without the N-terminal scaffold. The amino acid sequences are listed in Table 4. The flanking N-terminal phenylalanine was included where it stabilized the secondary structure according to the prediction tool.

SUPPLEMENTARY FIGURE 7 Peptide-mediated effect on the amyloidogenic properties of αSyn *in vitro*. Aggregation kinetics of αSyn in presence or absence of K50, K50s, K94s, or K97s monitored by ThT fluorescence emission. αSyn was incubated alone or upon the addition of the peptides at the indicated molar ratios. The ThT fluorescence emission was recorded every 15 min for 100 h. Incubation of the peptides alone served as a control.
