# Supplementary figures and images for: Identification of Two Novel Peptides That Inhibit α-Synuclein Toxicity and Aggregation
Source: Front Mol Neurosci. 2021 Apr 12;14:659926. doi: 10.3389/fnmol.2021.659926 (PMC8072481; doi:10.3389/fnmol.2021.659926)

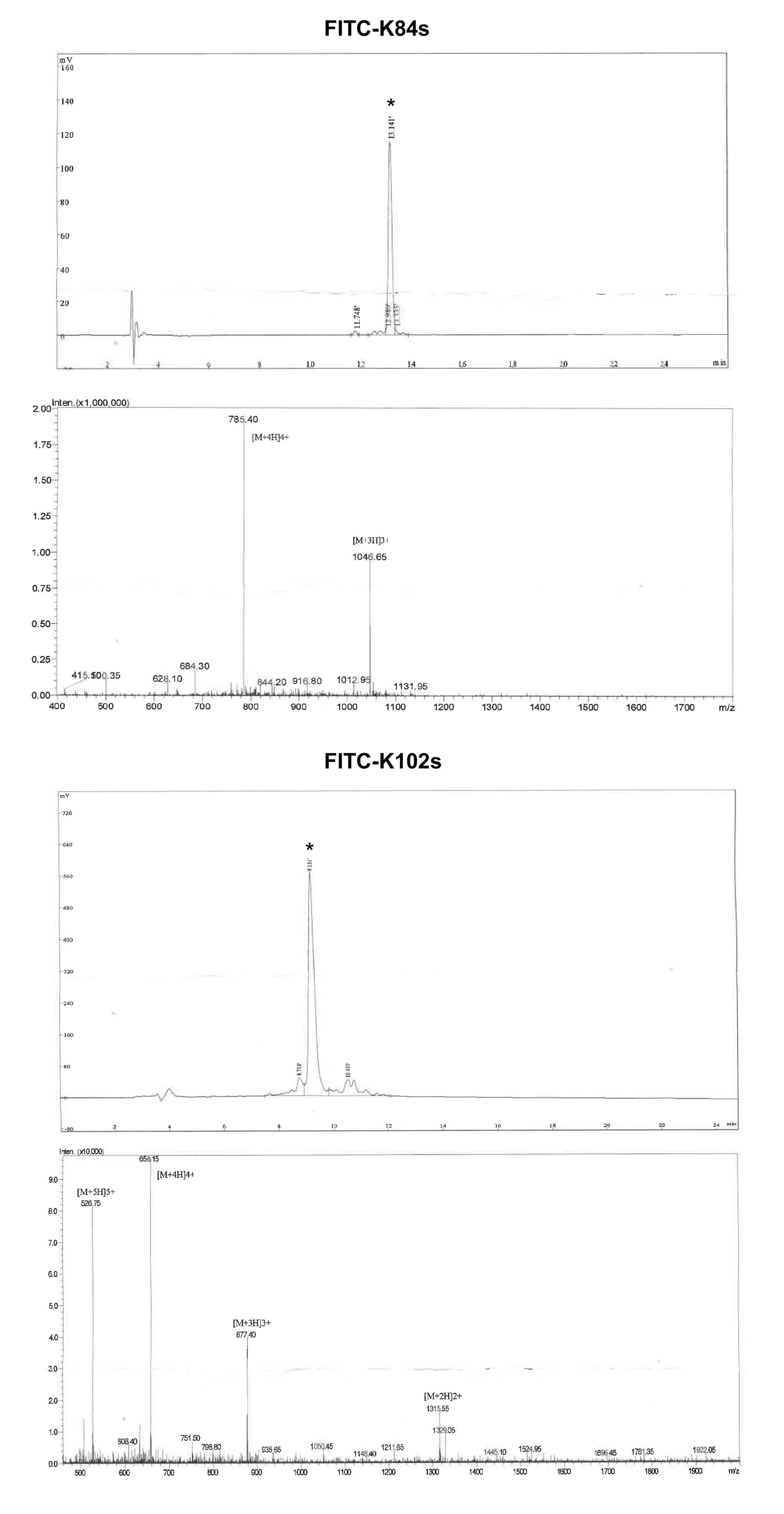

Supplement: FSUPPLEMENTARY FIGURE 1 — Analysis of peptides FITC-K84s and FITC-K102s. The upper panels represent the HPLC chromatograms of the samples. The lower panels represent the mass spectrometry spectrum of the sample peaks, marked with an asterisk in the upper panel. [file Image_1.TIF]

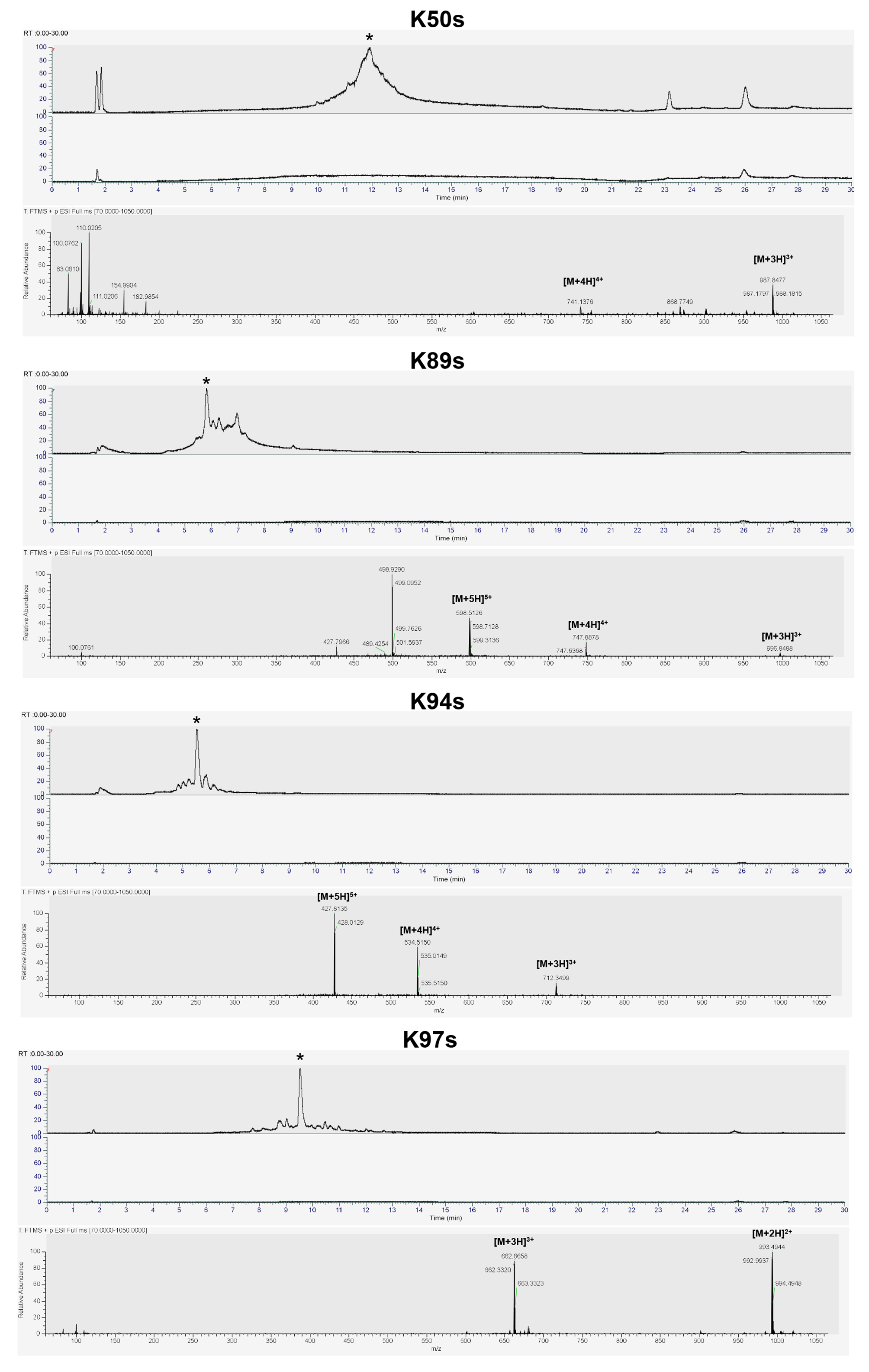

Supplement: SUPPLEMENTARY FIGURE 2 — Analysis of peptides K50s, K89s, K94s, and K97s. The peptides were analyzed with LC-MS equipped with a charged aerosol detector (CAD). The upper panel shows the CAD chromatogram of the peptide sample (above) compared to the solvent control (below). Y-axis gives the relative current in %. The lower panel shows the MS spectrum of the peptide peak, marked with an asterisk in the upper panel. [file Image_2.TIF]

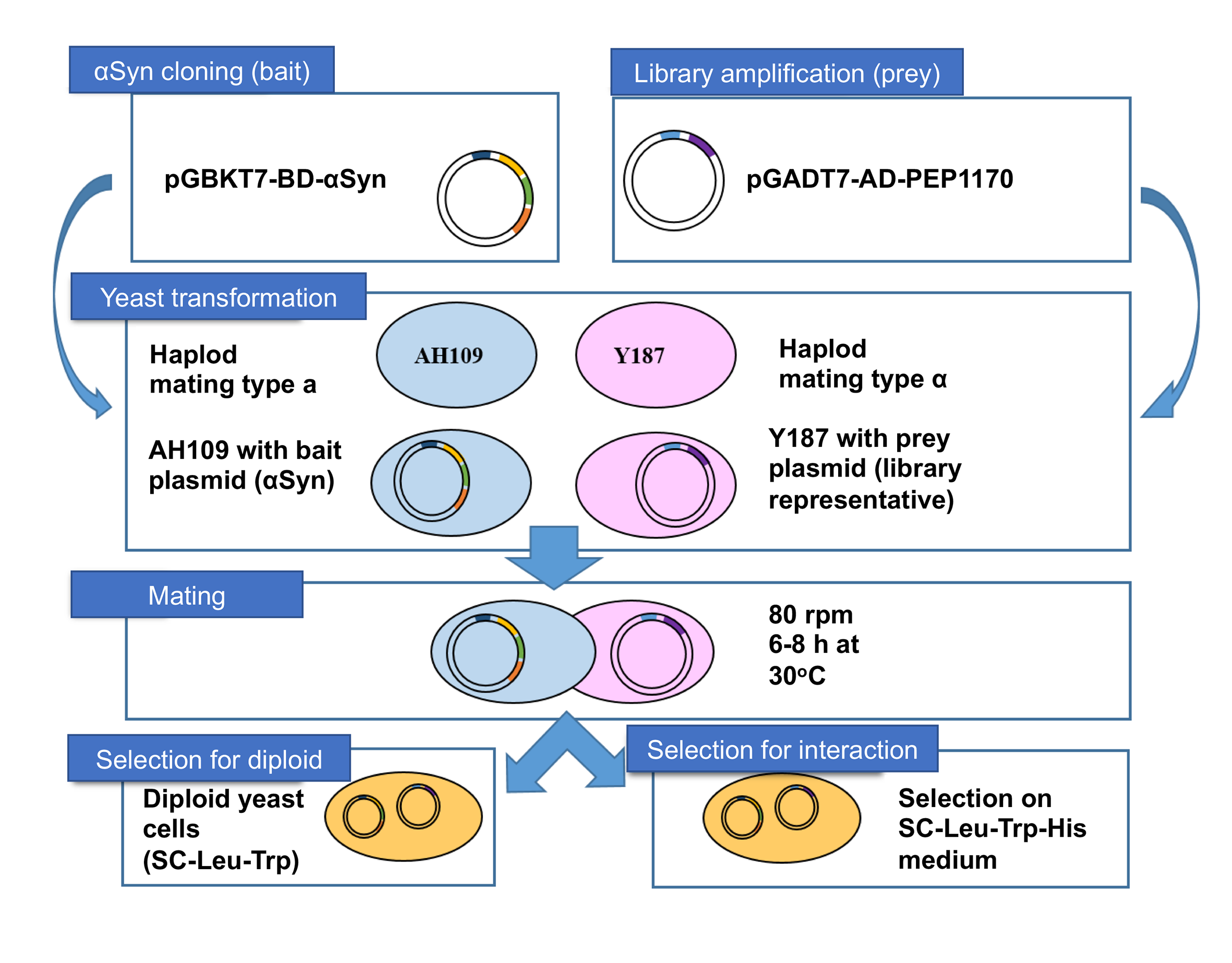

Supplement: SUPPLEMENTARY FIGURE 3 — Flowchart depicting yeast-two-hybrid (Y2H) screening. Y2H assay is a step-by-step procedure starting from the construction of bait (αSyn), fused to the GAL4 DNA-binding domain (BD) and amplification of the library, where each peptide construct is fused to B42 activation domain (AD). The bait is transformed into AH109 yeast strain that harbors HIS3 and ADE2 reporter genes driven by a GAL4 regulatory binding site to select for growth on media lacking histidine and adenine and was mated with the library. Selection for diploids is performed on SC-Leu-Trp medium. Positive clones are selected by their ability to activate the transcription of the reporter genes that enable growth on SC-Leu-Trp-His medium. [file Image_3.TIF]

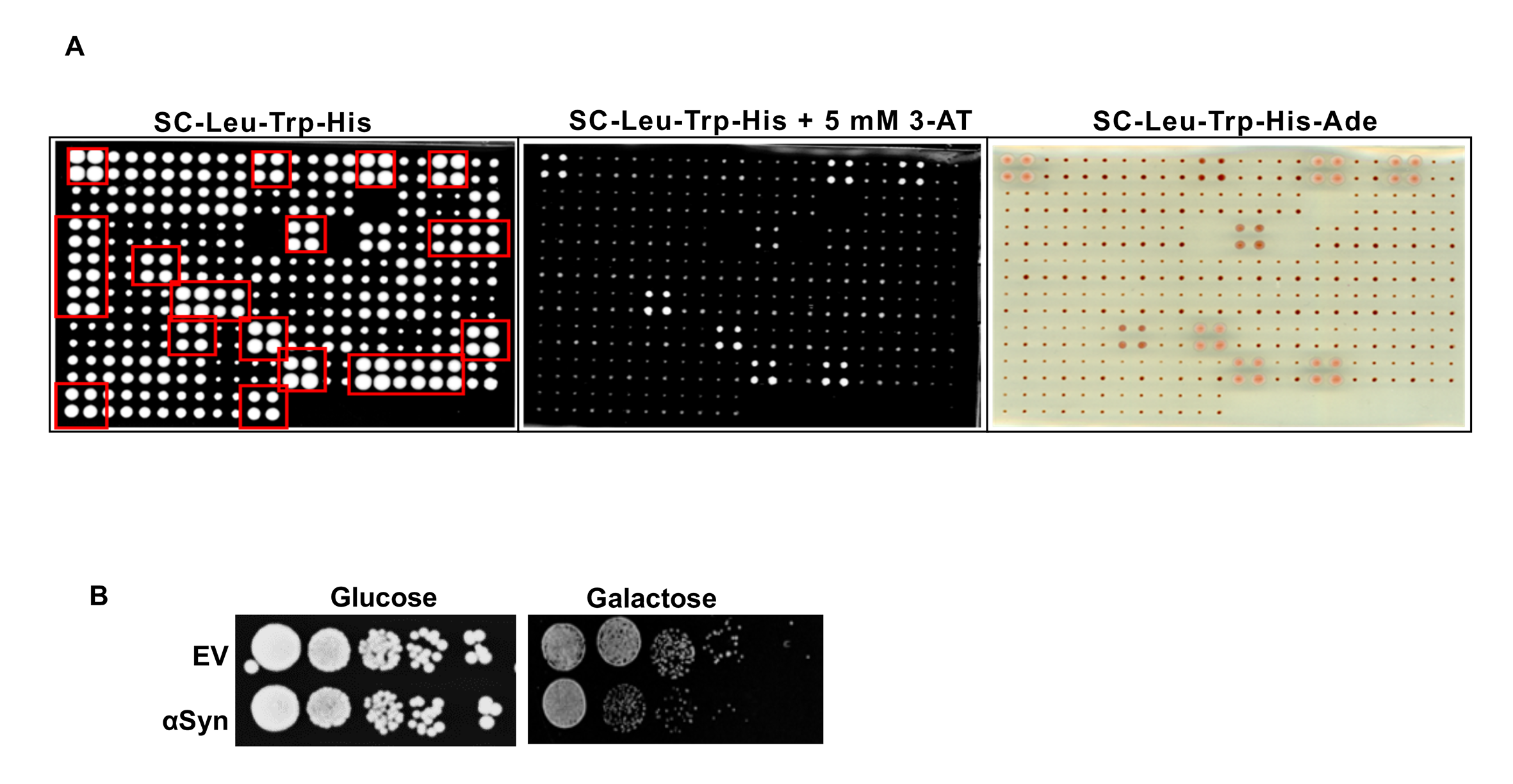

Supplement: SUPPLEMENTARY FIGURE 4 — Exemplary growth assay for selection of library candidates. (A) Ninety colonies were picked from the initial selection on SC-Leu-Trp-His semi-solid medium after 6 days of incubation at 30°C. The cells were spotted on SC-Leu-Trp plate, allowed to grow for 2 days, and pinned in four technical replicates onto agar selection plates with increasing stringency. Red boxes indicate clones, selected for plasmid isolation and sequencing. (B) Spotting assay of yeast strain expressing GAL1-driven αSyn used for the toxicity-rescue screen. Yeast strain AH109 was transformed with an integrative vector harboring αSyn-encoding gene or empty vector (EV) as a control. Yeast cells were spotted in 10-fold dilution on selective SC-Trp plates, containing 2% glucose or galactose and allowed to grow for 3 days at 30°C. [file Image_4.TIF]

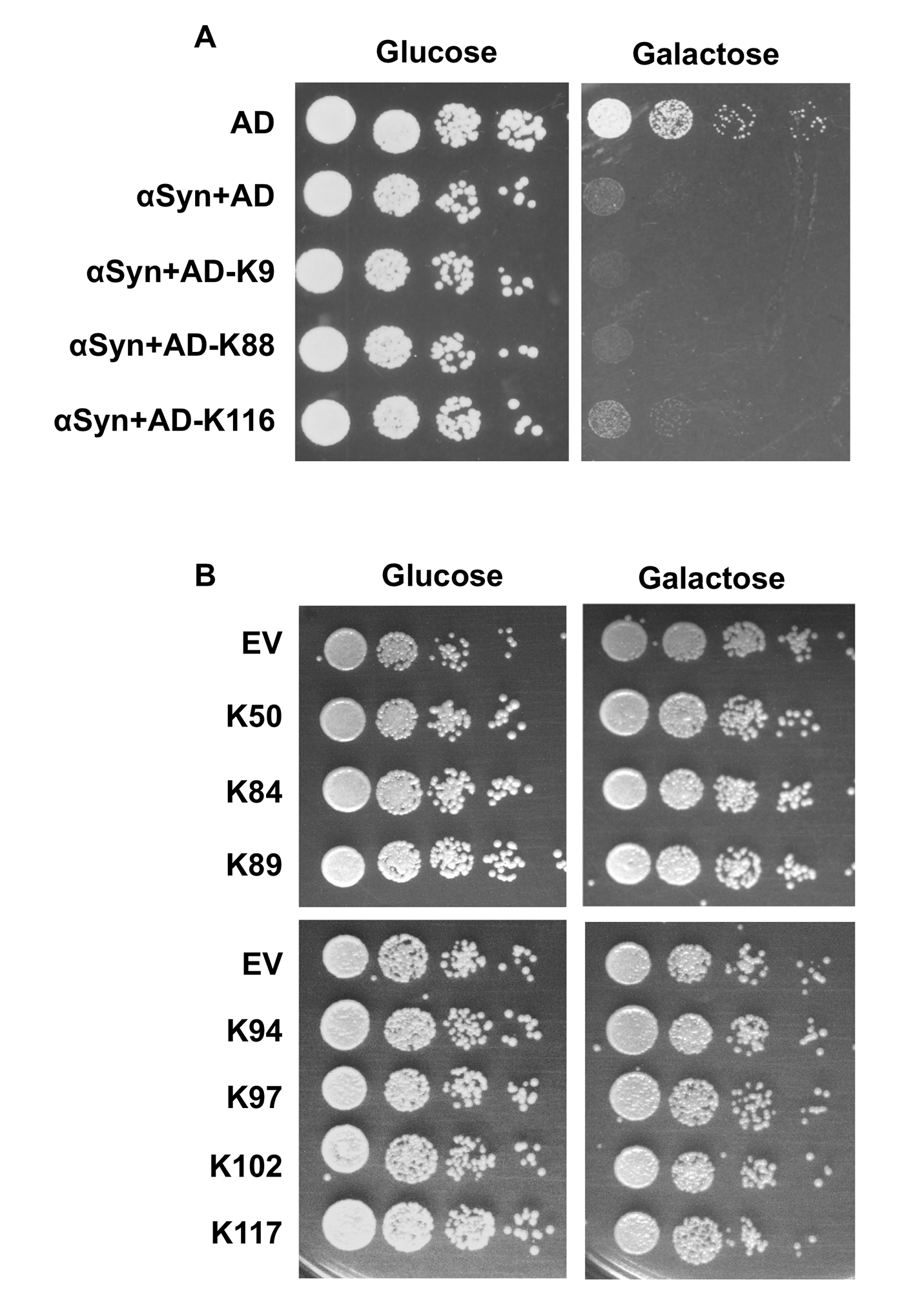

Supplement: SUPPLEMENTARY FIGURE 5 — Expression of the peptide constructs does not affect yeast growth. (A) Expression of random peptide constructs does not rescue αSyn-induced growth inhibition. A yeast strain that harbors three copies of the GAL1-driven αSyn-GFP-encoding gene was transformed with three random plasmid constructs that contain the peptide-encoding sequence fused to the B42-activation domain (AD), or with a vector with AD. Yeast cells were spotted in 10-fold dilution on SC-Leu-Ura selective plates containing glucose (control: αSyn “OFF”) or galactose (αSyn “ON”) for induction of GAL1 promoter. Yeast wild-type isogenic background strain transformed with AD-vector was used as positive growth control. (B) Yeast strain, expressing GAL1-driven GFP was transformed with the plasmids, encoding the peptide sequences without the N-terminal AD. Spotting assay was performed as described. Empty vector (EV) was used as a control. [file Image_5.TIF]

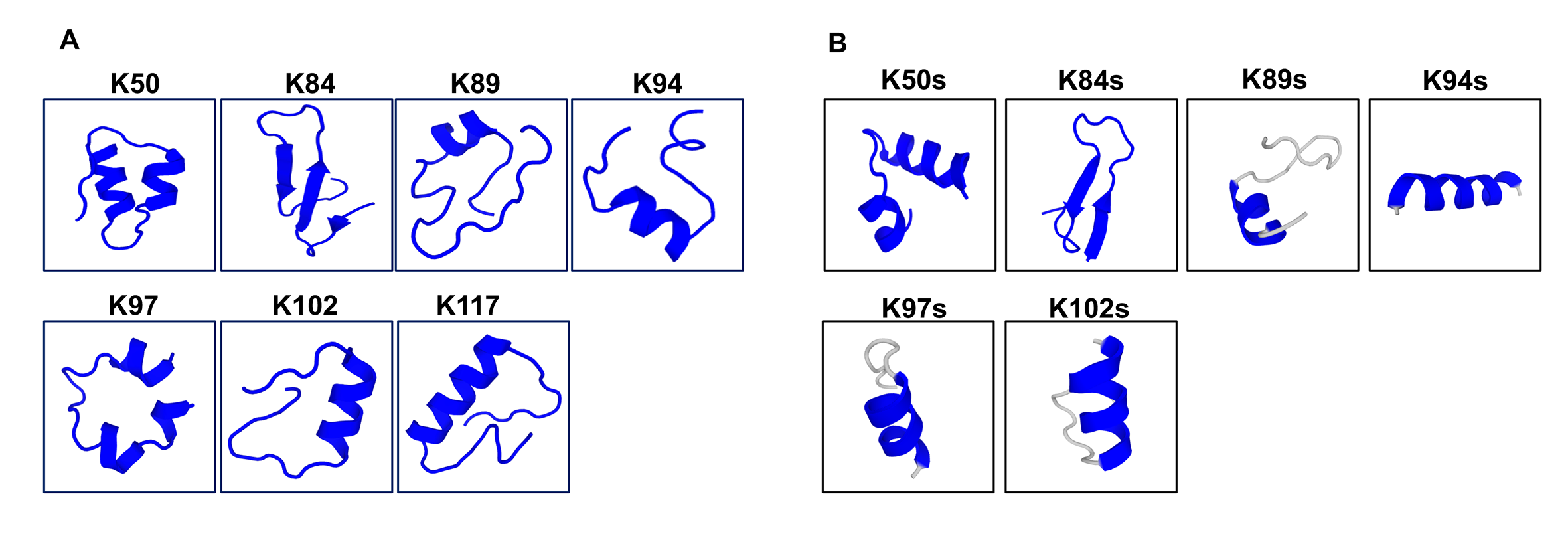

Supplement: SUPPLEMENTARY FIGURE 6 — Secondary structure predictions of the indicated peptides. PEP FOLD 3 tool was used for predicting peptide structures from amino acid sequences. (A) Peptide constructs with N-terminal GB1-scaffold expressed in yeast. K50 and K117 contain also the C-terminal scaffold sequence, as indicated in Table 3. (B) Predicted structure of the synthetic peptides consisting only of variable region sequences without the N-terminal scaffold. The amino acid sequences are listed in Table 4. The flanking N-terminal phenylalanine was included where it stabilized the secondary structure according to the prediction tool. [file Image_6.TIF]

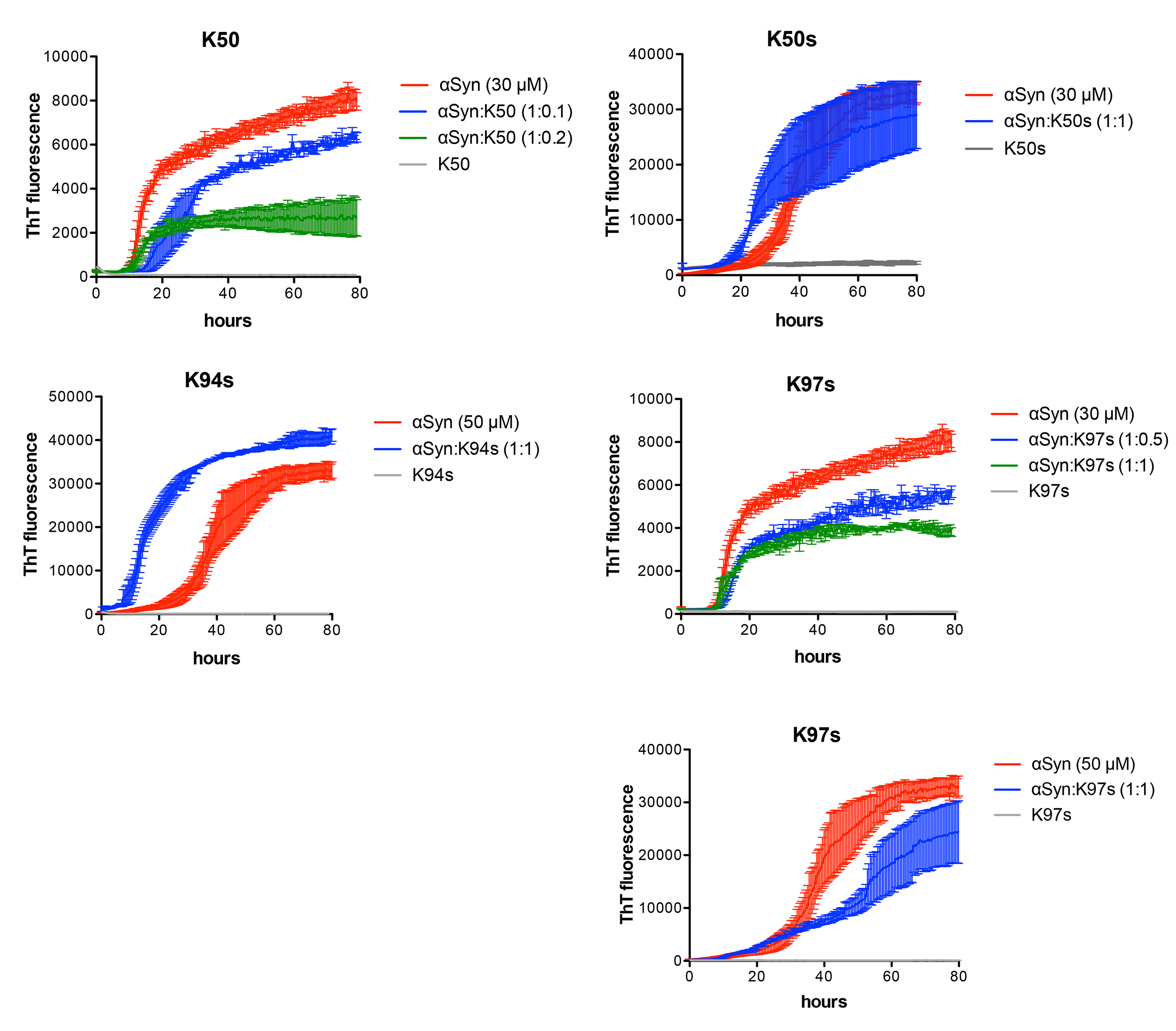

Supplement: SUPPLEMENTARY FIGURE 7 — Peptide-mediated effect on the amyloidogenic properties of αSyn in vitro. Aggregation kinetics of αSyn in presence or absence of K50, K50s, K94s, or K97s monitored by ThT fluorescence emission. αSyn was incubated alone or upon the addition of the peptides at the indicated molar ratios. The ThT fluorescence emission was recorded every 15 min for 100 h. Incubation of the peptides alone served as a control. [file Image_7.TIF]
